# Quality of Life and Mental Health in Caregivers of Children With Down Syndrome and Sleep Problems

**DOI:** 10.1111/jar.70103

**Published:** 2025-07-22

**Authors:** Kasey Fullwood, Andrew Collaro, Lachlan Power, Jasneek Chawla

**Affiliations:** ^1^ Child Health Research Centre, the University of Queensland South Brisbane Australia; ^2^ Department of Respiratory and Sleep Medicine Queensland Children's Hospital South Brisbane Australia

**Keywords:** caregiver mental health, down syndrome, paediatric, sleep

## Abstract

**Introduction:**

Children with Down Syndrome are more likely to experience sleep issues throughout their life compared to typically developing children. Sleep difficulties also affect caregivers, who are at increased risk of sleep disturbances, mood disturbances and poorer wellbeing. However, the impact of poor sleep in this cohort of children on their caregivers is not widely understood.

**Method:**

This study assessed the quality of life and mental health in 26 caregivers of children with Down Syndrome and sleep problems through two self‐reporting questionnaires.

**Results:**

Results showed caregivers had significantly lower quality of life (QoL) and higher stress scores compared to population norms. A decrease in psychological and physical health scores was associated with higher odds of depression. Similarly, a reduced physical and social health increased the odds of experiencing stress by 50%.

**Conclusion:**

These findings suggest this cohort of families may benefit from increased psychosocial support when addressing sleep problems.

**Abbreviations**
CPAP: continuous positive airway pressure
DASS: the Depression, Anxiety and Stress Scale
OSA: Obstructive sleep apnoea
QoL: Quality of Life
SDB: sleep disordered breathing
WHOQOLBREF: World Health Organisation Quality of Life Abbreviated Scale

**Summary**

- Caregivers of children with Down Syndrome with sleep issues have poorer QoL and mental health compared to the general population. This is relevant in clinical care given to children with Down Syndrome presenting with sleep issues and the wellbeing of their families.
- Sleep issues affect more than just the child not sleeping, and these effects can be detrimental to caregiver QoL and mental health.
- This study is one of the first to measure QoL and mental health in caregivers of children with Down Syndrome and sleep issues.
- Larger scale studies inclusive of all caregivers are needed to build upon our findings.

## 1 Introduction

Down Syndrome is the most common chromosomal disorder in humans, with a prevalence of approximately one in every 675 births worldwide (Hendrix et al. 2021). Compared to typically developing children, those with Down Syndrome are recognised to have a higher prevalence of sleep difficulties, encompassing both respiratory and non‐respiratory sleep problems (Churchill et al. 2012). Respiratory sleep problems encompass any abnormal respiration during sleep. Sleep disordered breathing encompasses both central and obstructive sleep apnoea. Whilst both may occur in children with Down Syndrome, obstructive sleep apnoea (OSA) is particularly prevalent in children with Down Syndrome (estimates range from 29% to 79%, compared to 1% to 5% in general paediatric population) (Marcus et al. 2012). OSA consists of complete and partial sleep apnoea and hypopnoea events which result in associated arterial oxygen desaturation and/or repeated arousals during sleep (Isaiah and Mitchell 2024). A combination of predisposing risk factors such as macroglossia, midfacial and mandibular hypoplasia, adenotonsillar hypertrophy, and hypotonia, coupled with other co‐morbid conditions such as obesity, increases their risk of OSA (Churchill et al. 2012; Maris et al. 2016). Behavioural sleep disorders such as bedtime resistance, sleep anxiety, night waking, and parasomnias have also been shown to commonly affect children with Down Syndrome (Hoffmire et al. 2014; Carter et al. 2009).

Treatments, including adenotonsillectomy and continuous positive airway pressure (CPAP) are commonly used to treat SDB in children with Down Syndrome; however, treatment tolerance and overall success vary significantly (Shete et al. 2010; Rosen 2011). Non‐respiratory sleep disorders can be even more challenging to manage. Pharmacological supplementation with melatonin has been shown to improve sleep onset in typically developing children; however, studies involving children with Down Syndrome are scarce and less conclusive (Braam et al. 2008; Ward et al. 2015). Behavioural interventions such as parental education and addressing sleep hygiene are also under‐researched for children with Down Syndrome.

Sleep disruption resulting from SDB or non‐respiratory sleep disorders have unique significance within this population due to associations with long‐term morbidity including cardiovascular disease and systemic inflammation, and the increased susceptibility of children with Down Syndrome to cardiovascular disease and metabolic disorders (Hanna et al. 2022; Gozal 2009; Nehme et al. 2017). Furthermore, sleep disorders contribute to a range of negative functional and cognitive outcomes in children with Down Syndrome. Children with Down Syndrome and recognised sleeping issues show poorer executive function, attention, language fluency and expression, and behavioural patterns than those without sleeping difficulties (Breslin et al. 2014; Chen et al. 2013; Edgin et al. 2015; Esbensen et al. 2018; Kelmanson 2017). These deficits may exacerbate pre‐existing intellectual disabilities that occur in this population and can lead to poorer overall outcomes (Churchill et al. 2012).

Families of children with Down Syndrome are also heavily affected by poor sleeping patterns and the follow‐on effects during waking hours. Carers of children with sleep issues are more likely to have sleeping difficulties of their own and associated mood disturbances (Varma et al. 2020). Our group recently published the first qualitative study to explore the impact that poor sleep in children with Down Syndrome can have on their caregivers and families, reporting largely negative experiences. Findings from this study suggest that caregivers of children with Down Syndrome understand that sleep disruption has adverse effects on their wellbeing and family dynamics; however, they are likely to normalise this poor sleep and the flow‐on effects (Chawla et al. 2023). This normalisation of sleep disorders in children with Down Syndrome can be exacerbated by inadequate or insensitive practices by healthcare professionals when addressing sleep‐related concerns in children with Down Syndrome (Chawla et al. 2023). These findings highlight considerable implications for the wellbeing and quality of life of those affected by sleep problems and represent the need for greater support from healthcare professionals. In this study, we report on psychosocial data from the caregivers who participated in the initial qualitative study. Our study aims were to evaluate the potential impact of their child's poor sleep on caregiver mental health and quality of life (QoL) by (i) comparing this cohort of caregivers' QoL and depression, anxiety, and stress scores against normative data and (ii) measuring the impact of caregiver mental health scores on their overall QoL.

### 1.1 Study Impact

Little is known about the impact of sleep disorders in children with Down syndrome on caregivers. This study provides preliminary data on the potential negative impact of sleep problems on caregivers' mental health and quality of life, highlighting this as an area that needs to be addressed in clinical practice as a priority by (i) ensuring sleep problems in children with Down syndrome are treated early, and (ii) with the provision of psychosocial supports for families.

## 2 Methods

### 2.1 Study Design

This study was undertaken as part of a larger mixed‐method study entitled *Sleep Difficulties in Children with Down Syndrome: An Evaluation of Parent/Carer and Family Quality of Life*. *The quantitative component, reported here, focused on caregiver mental health and quality of life using validated questionnaires. The qualitative component involved in‐depth interviews exploring parental experiences, perceived impacts of sleep difficulties, and support needs and has been reported separately* (Chawla et al. 2023; Cooke, Smith, et al. 2023; Cooke, Coles, et al. 2023). The study received ethical approval from the Children's Health Queensland HREC Committee. Approval Number: HREC/20/QCHQ/65571.

### 2.2 Participants

Twenty‐six caregivers of children under the care of the Sleep Unit at the Queensland Children's Hospital for a sleep problem who were enrolled in a concurrent qualitative study were included. Caregivers who participated in this concurrent qualitative study (Chawla et al. 2023) were asked to complete two questionnaires as part of the current study: the Depression, Anxiety and Stress Scale (DASS) and the World Health Organisation Quality of Life Abbreviated Scale (WHOQOLBREF) (Lovibond and Lovibond 1995a; Hawthorne et al. 2006). Caregivers were defined as those who care for children with Down Syndrome who had a diagnosed sleep disorder. Sleep disorders included respiratory and non‐respiratory sleep disorders, diagnosed by a respiratory and sleep specialist, based on clinical assessment, medical chart reviews, and sleep study findings (where appropriate). Once an interview date for the qualitative component of the study was scheduled, questionnaires were sent by mail to enrolled participants. Caregivers were reminded to complete the questionnaires when interviewed, and further prompts were provided by the research coordinator as needed for the following 3 months. Demographic and medical history data were collected using electronic medical records and a clinical history questionnaire.

### 2.3 Materials

#### 2.3.1 Depression and Anxiety Stress Scale

The DASS‐42 is a set of three self‐report scales designed to measure the negative emotional states of depression, anxiety, and stress (Lovibond and Lovibond 1995b). It is a validated measure of mental health in adults and consists of 42 questions. Normative data for this assessment were developed from a sample of 1044 males and 1870 females aged between 17 and 69 years of varying social backgrounds. Scales for surveyed family members were compared to normative means and 95% confidence intervals (95% CIs). Higher scores on the DASS‐42 represent higher rated depression, anxiety, and stress. Scores are represented on a scale of Normal to Severe.

#### 2.3.2 World Health Organisation Quality of Life Abbreviated Scale

The WHOQOL‐100 assessment was developed by the WHOQOL group with 15 international field centres to develop a QoL assessment that would be applicable cross‐culturally and has been previously used in studies involving families of children with Down Syndrome. The WHOQOLBREF is an abbreviated version of this questionnaire which is based on a four‐domain structure (physical health, psychological, social relationships, and environment). It is a self‐administered, validated questionnaire that consists of 26 questions. The questionnaire produces a QoL profile which includes four domain scores, which denote an individual's perception of QoL in each domain, as well as two individual questions that ask about an individual's overall perception of QoL (Q1) and an individual's overall perception of health (Q2). Raw domain scores are transformed to a scale of 0–100, and scores for surveyed family members were compared to all‐age mean values and 95% CIs (WHOQOL 1996). Higher scores in each domain represent better quality of life in those health domains.

### 2.4 Data Analysis

Descriptive statistics including means and standard deviations (SDs) were presented for all four domains of the WHOQOLBREF and the DASS. Participant scores were compared to normative data using *T*‐tests for each WHOQOLBREF domain. Participant age was not recorded; participant scores were compared to all‐age values. *T*‐tests were done on Depression, Anxiety, and Stress scores against normative data (Lovibond and Lovibond 1995a). Pearson's correlation matrix was used to capture correlations between QoL and DASS scores, along with intercorrelations between demographic variables. Univariable logistic regression was used to determine whether QoL scores could predict mild, moderate, or severe depression, anxiety, and stress.

### 2.5 Demographic Data

Twenty‐six of the 34 caregivers (76.5%) who participated in the concurrent qualitative study returned completed DASS and WHOQOLBREF questionnaires. Demographic characteristics of study participants are summarised in Table 1.

**TABLE 1:** Demographic information for 26 participants.

	Sub‐category	Number of participants (*n* = 26, including 24 females)
Family structure	Nuclear (two caregiver household)	19 (73%)
	Separated with dual custody	4 (15%)
	Single caregiver	3 (12%)
Siblings in the family	Yes	12 (46%)
	No	14 (54%)
Primary caregiver education level	Tertiary	19 (73%)
	Up to year 11/12 (secondary education)	7 (27%)
	Less than year 11/12 (secondary education)	0
Primary earner occupation type	Skilled/professional	15 (58%)
	Semi‐skilled	9 (35%)
	Unskilled	2 (8%)
Employment status if primary earner	Full‐time	11 (42%)
	Part‐time	12 (46%)
	Unemployed/pension	3 (12%)

## 3 Results

**FIGURE 1:**
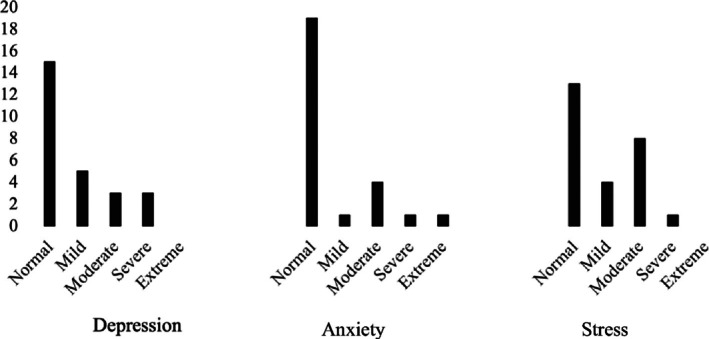
Histograms of Depression, Anxiety and Stress scores for 26 study participants.

Environment health scores were the highest of the four QoL domains among carers with children with Down Syndrome, with Social health the lowest (Table 2). On average, caregivers' Stress scores were highest on the DASS scale, followed by Depression and Anxiety (Table 3). Mean depression and anxiety scores were within the normal range, with stress mild in participants. Further analysis showed 50% of stress scores, 42.3% of depression scores, and 26.2% of anxiety scores in the mild–extreme ranges. Of these, 11.5% of participants rated their depression as severe, whilst 38% showed severe stress scores (Figure 1).

**TABLE 2:** Mean scores, mean difference to normative data, *t*‐scores and significance levels of quality of life scores.

	Mean (SD)	Mean difference	*t*‐score	*p*‐value
Physical health	58.34 (14.54)	15.15	−4.24	< 0.001
Psychological health	56.5 (14.99)	14.1	−5.05	< 0.001
Social health	49.03 (24.21)	22.46	−6.13	< 0.001
Environment health	67.96 (15.56)	7.14	−2.74	0.006

**TABLE 3:** Mean scores, mean differences, *t*‐scores and significance levels of participant Depression, Anxiety and Stress Scale scores compared to normative values (Lovibond and Lovibond 1995a).

	Mean (SD)	Mean difference	*t*‐score	*p*‐value
Depression	8.46 (7.46)	2.12	1.543	0.123
Anxiety	5.77 (6.83)	1.07	1.101	0.271
Stress	14.77 (7.47)	4.66	2.9921	0.003

### 3.1 Comparison to Normative Data

When comparing participant QoL scores for each domain against normative data, all domains of health were significantly lower than the normative data (WHOQOL 1996) (Table 2).

When compared to DASS normative data (Lovibond and Lovibond 1995a), participants had a higher mean stress score, whilst depression and anxiety scores of participants were not significantly different from normal values (Table 3).

### 3.2 Correlations

Pearson's correlation values outline relationships between all variables (Table 4). Depression was negatively correlated to all QoL variables. Further, depression showed a significant positive correlation to anxiety and stress, where anxiety and stress scores increased as depression scores increased. Psychological health was significantly positively correlated with social relationships and environment. Further, physical health was significantly negatively correlated with stress. All other variables showed non‐significant correlations. No significant differences were found between demographic variables.

**TABLE 4:** Correlation values for four quality of life domains and Depression, Anxiety and Stress Scale scores.

	Physical	Psychological	Social relationships	Environment	Depression	Anxiety	Stress
Physical health	—						
Psychological	0.31	—					
Social relationships	0.03	0.43*	—				
Environment	0.07	0.56**	0.55**	—			
Depression	−0.58**	−0.63***	−0.45*	−0.49*	—		
Anxiety	−0.34	−0.10	−0.35	−0.32	0.56**	—	
Stress	−0.46*	−0.23	−0.51	−0.33	0.58**	0.67**	—

*Note:* *p < 0.05; **p < 0.01; ***p < 0.001.

### 3.3 Logistic Regression

#### 3.3.1 Depression

**FIGURE 2:**
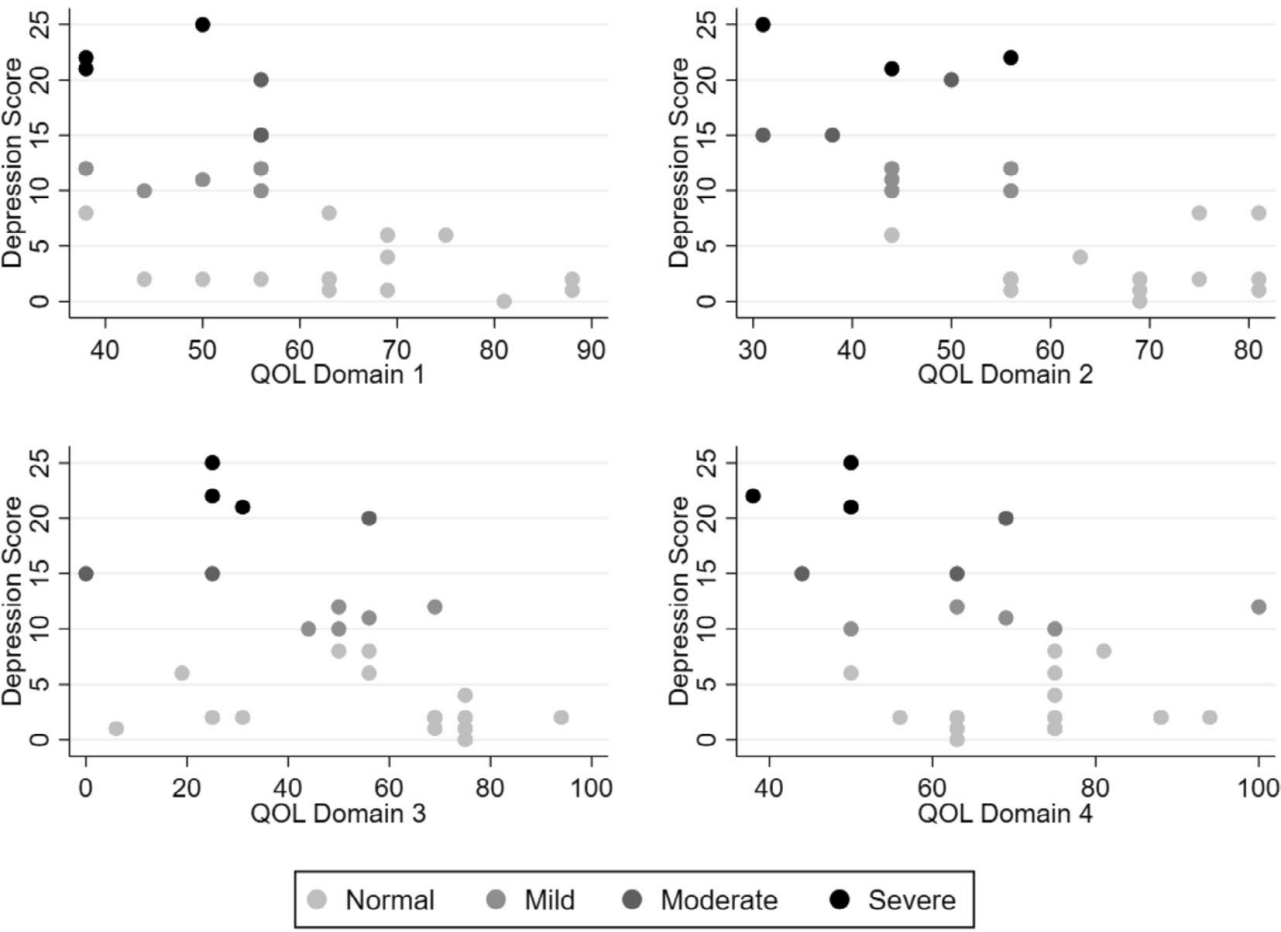
Scatter plots of depression scale scores against World Health Organisation Quality of Life Abbreviated Scale scores. Shading indicates level of depression as determined by Depression, Anxiety, and Stress Scale.

Each single point reduction in QoL for physical health (odds ratio [OR] = 0.88, 95% CI 0.78–0.98, *p* = 0.016) and psychological health (OR = 0.84, 95% CI 0.74–0.96, *p* = 0.011) was associated with higher odds of reporting mild, moderate, or severe depression (Figure 2). Therefore, participants with decreased physical and psychological health were more likely to report symptoms consistent with mild–severe rated depression. There was no statistical significance for social relationship health (OR = 0.97, 95% CI 0.93–1.00, *p* = 0.084) and environment health (OR = 0.88, 95% CI 0.69–1.00, *p* = 0.063).

#### 3.3.2 Anxiety

There was no statistical significance for all four domains on anxiety, with QoL scores not associated with anxiety scores.

#### 3.3.3 Stress

**FIGURE 3:**
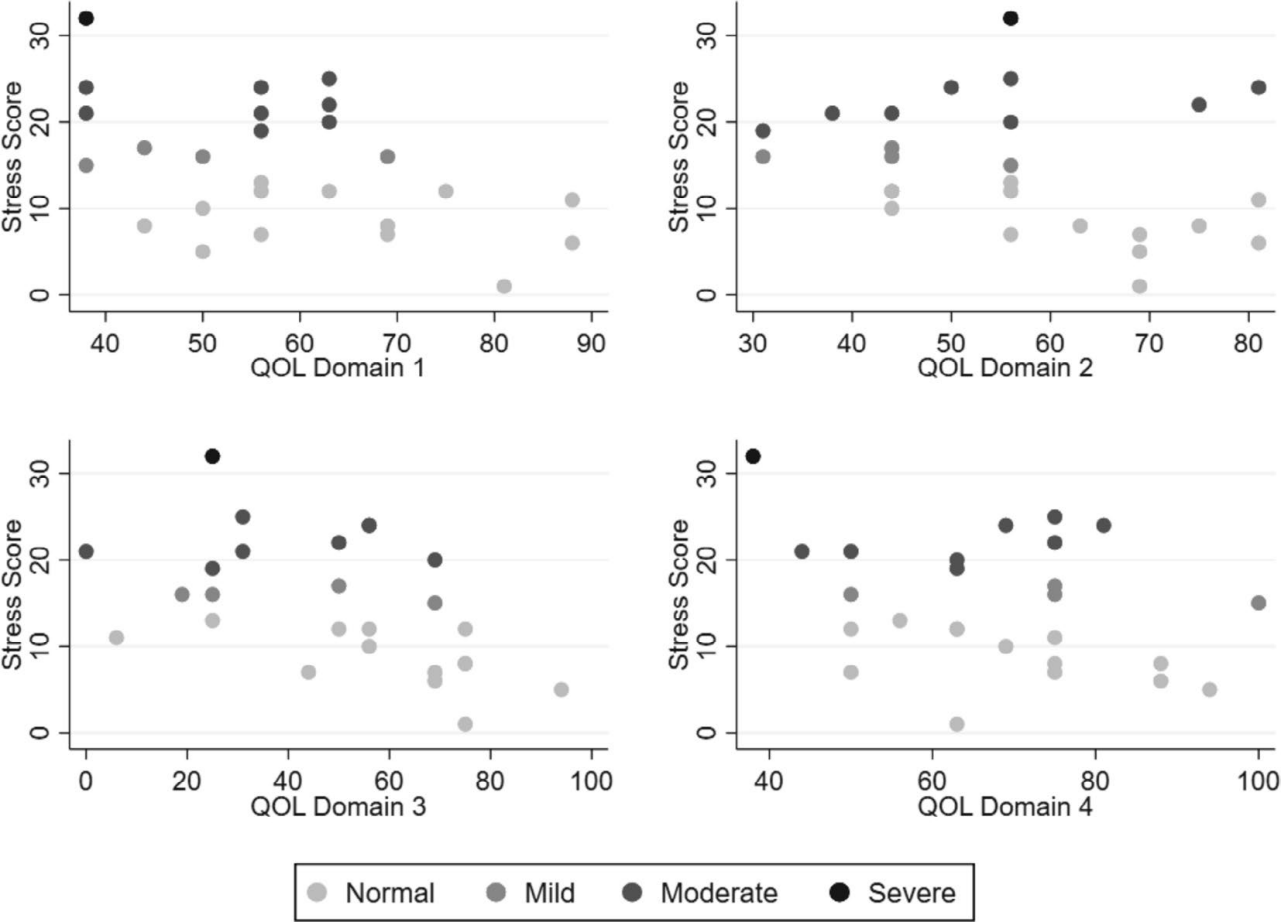
Scatter plots of stress scale scores against World Health Organisation Quality of Life Abbreviated Scale scores. Shading indicates level of stress as determined by Depression, Anxiety, and Stress Scale.

Each single point reduction in QoL for physical health (OR = 0.92, 95% CI 0.85–0.99, *p* = 0.033) and social relationship health (OR = 0.96, 95% CI 0.92–0.99, *p* = 0.043) was associated with increased odds of reporting mild/moderate/severe stress by 50% (1.5) (Figure 3). Therefore, participants with lower physical and social health were more likely to report mild–severe levels of stress. No statistical significance was found for psychological (OR = 0.94, 95% CI 0.89–1.00, *p* = 0.067) and environment health (OR = 0.98, 95% CI 0.93–1.03, *p* = 0.515).

## 4 Discussion

In this study of 26 caregivers of children with Down Syndrome who had recognised sleep issues, we found lower QoL scores across multiple health domains when compared to the general population, along with higher stress scores. Further, we found low QoL predicted the occurrence of increased depression and stress scores. These results begin to outline the impact of sleep issues on caregivers with children with Down Syndrome. Existing literature outlines the profound negative effect sleep has on parental mental health and quality of life in typically developing children and other developmental disorders (Varma et al. 2020; Merrill and Slavik 2023; Abdullah et al. 2021; Martin et al. 2021; Liu et al. 2021); however, to our knowledge, this is one of only two studies to show these effects in caregivers of children with Down Syndrome. In their study of QoL in a Korean group of caregivers of children with Down Syndrome, Choi et al. found that sleep issues in children with Down Syndrome had a negative impact on participants (Choi et al. 2019). However, sleep was not the primary focus of this study, and it did not specifically examine a cohort of children with Down Syndrome who had confirmed sleep disorders. A systematic review and meta‐analysis on the QoL of family caregivers of children and young adults with Down Syndrome highlighted lower QoL when compared to population norms (Cheok et al. 2024). Authors reported various contributing factors to reduced QoL, including poor sleep quality in the child, outlining a consistent pattern within the literature of poor sleep impacting QoL in caregivers. More recently, a study evaluating patient and carer priorities of children with Down Syndrome found carers identified sleep quality as one of the most important research and clinical care priorities (Taylor et al. 2025). These studies add to the growing body of literature on the perceived importance of child sleep from caregivers and how poor sleep in the child can have flow‐over effects to caregivers, especially within the Down Syndrome population.

In this study we found that within QoL, lower physical, psychological, and social relationship health was observed in caregivers compared to reference norms, suggesting that caring for a child with Down Syndrome who is experiencing sleep problems may contribute to lower QoL. Caregivers stress was found to be higher than the general population, suggesting that the presence of a child with Down Syndrome who is experiencing sleep issues may contribute to a more stressful household. Other mental health scores of depression and anxiety did not differ from population norms. This could be attributed to the mental health measures having lower sensitivity to smaller sample sizes, failing to capture population differences. These findings highlight an important issue for clinicians, demonstrating the importance of addressing caregiver sleep and mental health alongside the child with Down Syndrome's sleep difficulties. A lack of sleep can have flow‐on effects to many aspects of a caregiver's life, from experiencing their own diminished sleep to caring for tired and frustrated children. Holistic evaluation of a family's sleep may in fact be vital to achieve success in treating the child with Down Syndrome's sleep—unless the caregiver is supported, the implementation of strategies to improve sleep in the child is unlikely to be successful. We have recently demonstrated the impact on siblings of children with Down Syndrome who sleep poorly, further emphasising the need to address the needs of entire families when managing sleep in a child with Down Syndrome (Cooke, Smith, et al. 2023).

As other studies have shown (Hohls et al. 2021; de Freitas et al. 2023), there is some correlation between quality of life and depression. For example, caregivers who reported low mood and had high depression scores were found to have poorer QoL. Similarly, lower QoL in specific health domains correlated with clinically significant levels of depression and stress. The previously published qualitative data from the same cohort by our group supports that poor sleep in the child with Down Syndrome may contribute to negative impact on psychosocial wellbeing in these caregivers and highlights again, that providing psychological support for caregivers when they present with a child with sleep disruption is an important part of management to consider (Chawla et al. 2023). Unlike these other studies, we did not observe any significant correlation between QoL and anxiety (Hohls et al. 2021; de Freitas et al. 2023), which could be attributed to our smaller sample size.

There were some limitations to this study. Whilst participants were caregivers of children with Down Syndrome who had sleep issues, we did not correlate the severity of sleep problems in the child with Down Syndrome with carer mental health and QoL scores. Therefore, we cannot definitively conclude there is an association between poor sleep and worse parental outcomes. The small number of participants in this study does considerably limit our ability to draw definitive conclusions regarding the relationship between poor sleep in a child with Down Syndrome and caregiver mental health and QoL. Our smaller sample size may also increase Type II errors, leading to results not reaching statistical significance. Most caregivers in this study were female and therefore we cannot comment on whether findings would be similar in male caregivers. Similarly, as is often the case in research, our study attracted a particular demographic of participants, and our sample consisted largely of nuclear families reflecting a two‐caregiver household. As such, this may not accurately represent all caregivers' mental health and QoL. We hypothesise that caregivers from single‐parent households may in fact have worse outcomes due to being the sole caregiver responsible for their child with Down Syndrome. Further, we did not have a control group of children with Down Syndrome who did not have sleep problems. This was overcome by undertaking comparison to reference values and we recognise this is a less robust method than recruiting a control group from within the same community. Finally, it is also important to acknowledge the potential for bidirectional relationships, whereby poor quality of life may contribute to psychological distress, and conversely, symptoms of depression or stress may negatively influence perceptions of quality of life. However, our recently published concurrent qualitative study supports a negative association between sleep disruption in a child with Down Syndrome and parental outcomes and describes in more detail the negative experiences of individual caregivers (Chawla et al. 2023).

With these limitations in mind, our results can only infer the potential impact sleep issues may have on caregivers QoL and mental health. This relationship is however supported by studies in children with other developmental disorders such as Attention Deficit and Hyperactivity Disorder (Martin et al. 2021) and Autism Spectrum Disorder (Abdullah et al. 2021; Liu et al. 2021), as well as in typically developing children (Varma et al. 2020; Merrill and Slavik 2023). Further research involving a larger number of participants is necessary to confirm the exact nature of the relationship between poor sleep in children with Down Syndrome and parental outcomes. Furthermore, comparing these findings to data from caregivers of children with other health care needs would provide valuable insight into whether the outcomes observed are specific to parenting a child with Down syndrome or reflect broader experiences associated with caregiving for children with complex developmental or medical conditions.

However, based on the initial findings in this study, we recommend that clinicians consider the need to explore family wellbeing and mental health when evaluating a child with Down Syndrome who presents with sleep difficulties. Integrating screening for caregiver mental health and quality of life allows early identification of those needing additional supports. Alongside evaluating the family wellbeing, clinicians should integrate caregiver supports when treating sleep in the child. These could be in the form of clinical resources, specific sleep management programmes, such as CBT‐I, community programmes or peer support networks. Future research should focus on developing such resources and programmes to allow children with Down Syndrome management of their sleep problems. For policymakers, this study emphasises the importance of embedding caregiver supports within healthcare, through existing or new government disability schemes. This could ensure respite or financial subsidies for care are available to caregivers to avoid the escalation of caregiver burden and may help to reduce the impact on their health and wellbeing. Future studies focusing specifically on the economic burden associated with providing care for children with sleep problems would additionally assist in advocating for improved resources in caregivers.

## Ethics Statement

The study received ethical approval from the Children's Health Queensland HREC Committee. Approval Number: HREC/20/QCHQ/65571. Participant consent was obtained in a concurrent qualitative study (Chawla et al. 2023).

## Conflicts of Interest

The authors declare no conflicts of interest.

## Data Availability

The data that support the findings of this study are available on request from the corresponding author. The data are not publicly available due to privacy or ethical restrictions.
